# Stress in Family Caregivers of Children with Chronic Health Conditions: A Case–Control Study

**DOI:** 10.3390/children11111347

**Published:** 2024-11-02

**Authors:** Jaqueline Brosso Zonta, Aline Cristiane Cavicchioli Okido, Bruna Josiane de Lima, Bianca Annie Martins, Wendy Sue Looman, Luis Carlos Lopes-Júnior, Fernanda Machado Silva-Rodrigues, Regina Aparecida Garcia de Lima

**Affiliations:** 1 Department of Nursing, Federal University of São Carlos (UFSCar), São Carlos 13565-905, Brazil; jaquelinezonta@estudante.ufscar.br (J.B.Z.); brunajosiane@estudante.ufscar.br (B.J.d.L.); biancaannie@estudante.ufscar.br (B.A.M.); 2 School of Nursing, University of Minnesota (UMN), Minneapolis, MN 55455, USA; looma003@umn.edu; 3 Department of Nursing, Federal University of Espirito Santo (UFES), Vitoria 29047-105, Brazil; luis.lopes@ufes.br; 4 School of Nursing, University of Sao Paulo (USP), São Paulo 05403-000, Brazil; fermachado@usp.br (F.M.S.-R.); limare@eerp.usp.br (R.A.G.d.L.); 5 Ribeirão Preto College of Nursing, University of Sao Paulo (USP), Ribeirao Preto 14040-902, Brazil

**Keywords:** disabled children, family caregivers, stress, salivary cortisol

## Abstract

Objectives: This study analyzed the stress experienced by family caregivers of children with special healthcare needs and identified associated factors. Methods: A case–control study was conducted with the “cases” being caregivers of children with chronic conditions and the “controls” being caregivers of healthy children. Recruitment was carried out via social media and complemented by snowball sampling. A sociodemographic questionnaire and the Perceived Stress Scale were applied, with four saliva samples collected in one day (at 8:00 am, 30 min later, and 4 and 12 h later). The Area Under the Curve for total daily cortisol production was calculated using the log-trapezoidal method. The Wilcoxon test and repeated-measures ANOVA were used for statistical analysis. Results: In total, 100 caregivers participated, with 50 in the “case” group and 50 in the “control” group. Significant differences in stress scores and salivary cortisol levels were observed between the groups, with the other variables constant. In both groups, cortisol levels followed a typical circadian pattern. Family income was associated with perceived stress. Caregiver age significantly explained perceived stress (*p* = 0.0098) and total cortisol production. Caregiver occupation also influenced cortisol results. Conclusions: Caregivers of children with chronic conditions showed higher perceived stress and lower cortisol production compared to those of healthy children. Family income, occupation, and caregiver age were associated with stress.

## 1. Introduction

Chronic health conditions have a biological, psychological, or cognitive origin and persist for at least one year or almost certainly as long. Children with chronic health conditions may experience limitations in functioning, activities, or social roles compared to healthy peers of the same age. In general, they rely on medications, specialized diets, medical technology, assistive devices or personal support. Additionally, they may need medical care, psychological services, or educational support that exceed the typical requirements for a child of that age or ongoing treatments, interventions, or accommodations either at home or in school [1].

Family caregivers of children with chronic health conditions are predisposed to experiencing elevated levels of stress due to the cumulative burden of caregiving responsibilities, uncertainties regarding prognosis, financial impact, and feelings of guilt, particularly in cases involving hereditary conditions or trauma [2]. Some family members, often mothers, provide highly specialized care for up to 24 h a day, which impacts sleep duration and quality, resulting in excessive fatigue and depressive symptoms [3]. These caregivers also exhibit significantly higher rates of depression, anxiety, and referrals to secondary mental health services compared to families of children without long-term illnesses and an increased incidence of obesity, hypertension, and back pain [4]. They are subjected to elevated levels of psychological stress and mental strain, with an increased risk of family dysfunction and disruption [5].

Among the systems that regulate stress levels, the hypothalamic–pituitary–adrenal (HPA) axis plays a prominent role, inducing alterations in cortisol levels [6]. Prolonged elevated cortisol levels can lead to suppressed immune responses and increased susceptibility to chronic health conditions among caregivers [7], as well as heightened risks of cardiovascular diseases, autoimmune disorders, and chronic inflammatory conditions [8].

Despite robust evidence supporting a physiological mechanism of stress, many stress measurements in the literature still rely exclusively on self-report measures. When used in isolation, these measures are limited due to cultural differences in assessment, reliance on conscious recall bias, variability in experiences over time, and calibration effects [9]. Therefore, the integration of biomarkers and self-report measures recognizes the need to adopt a complex system perspective to better understand the dynamic interactions between stress, social contexts, and caregiving outcomes [10]. Additionally, stressors may be perceived differently across individuals, making it crucial to identify the sociodemographic and psychosocial factors that contribute to positive adaptation during a child’s prolonged diagnosis and treatment [11].

In light of these considerations, the objective of the present study was to analyze the stress experienced by family caregivers of children with chronic health conditions and to identify the associated factors.

Our research was guided by the following hypotheses:

(1) Family caregivers of children with chronic health conditions experience higher levels of perceived stress.

(2) Family caregivers of children with chronic health conditions exhibit a higher total daily production of salivary cortisol.

(3) Significant correlations exist between sociodemographic variables and both perceived stress levels and salivary cortisol.

## 2. Materials and Methods

This is a case–control study [12] conducted in the state of São Paulo, Brazil. Family members aged 18 years or older who were primarily responsible for the care of children with chronic conditions were eligible for the case group. For the recruitment of the control group, dyads (caregiver/child) were matched by sex and age in a 1:1 ratio, with a maximum age difference of 10 years for caregivers and 2 years for children. The exclusion criterion for both groups was the use of glucocorticoid medications. No type of reward or payment was offered to the participants.

Prior to the start of data collection, a training session was conducted to align and standardize the procedures in order to minimize potential collection bias. The laboratory team conducted this training. Data collection took place between March 2022 and June 2023 and was conducted by three trained researchers. Recruitment was carried out through the distribution of flyers on social media platforms. Additionally, the “snowball” sampling strategy was employed [13].

On the day prior to the scheduled data collection, participants received a text message with preparation guidelines, which included refraining from eating or drinking anything one hour before collection, avoiding brushing teeth or using mouthwash before collection, abstaining from smoking or exercising one hour before collection, and avoiding alcohol consumption for 24 h prior to collection.

**Figure 1:**
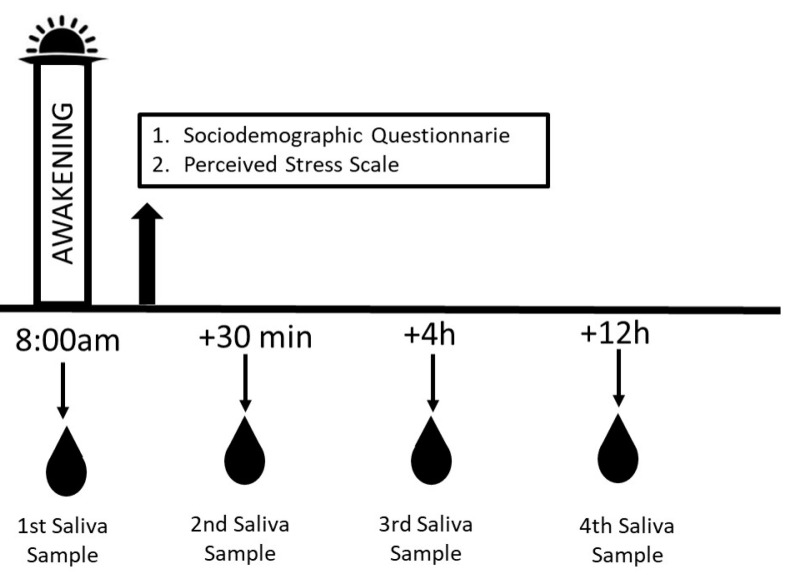
Data collection protocol.

The data collection protocol is illustrated in Figure 1. For the collection of salivary cortisol, the Salivette^®^ device (SARSTEDT AG & Co. KG, Nümbrecht, Germany) was used, which consists of an absorbent swab that participants gently chewed for 2 min to obtain a minimum saliva volume of 500 µL. Salivary cortisol concentrations were determined using the electrochemiluminescence method (Elecsys 1010/1020 analyzers, Roche Diagnostic, Laval, QC, Canada). Cortisol levels were reported in the units of µg/dL.

A sociodemographic characterization instrument was administered to collect data on the family caregiver’s age, education level, marital status, and occupation, the household income, and the child’s age. Additionally, the Perceived Stress Scale (PSS) was utilized, which consists of 14 questions with Likert-type response options (0 = never; 1 = almost never; 2 = sometimes; 3 = almost always; 4 = always). The total score on the scale ranges from 0 to 56, with higher scores indicating greater perceived stress. This scale also assesses stressful situations encountered in the past 30 days, e.g., “In the last month, how often have you felt that things were going your way?” and “In the last month, how often have you been able to control irritations in your life?” The instrument demonstrates high internal consistency, with Cronbach’s α values 0.84 in the original study [14] and 0.82 in the Brazilian version [15].

The stress measured by the Perceived Stress Scale score and salivary cortisol levels was considered the dependent variable. The sociodemographic variables of interest included the family caregiver’s age, education level, marital status, and occupation, the household income, and the child’s age. Cortisol levels were analyzed based on the values obtained at each collection time and total daily production. To determine daily production, the Area Under the Curve with respect to ground (AUCG) was calculated using the log-trapezoidal method [16].

For statistical analysis, the Wilcoxon test was employed, which is a comparison test suitable for quantitative variables that do not follow a normal distribution. Additionally, a repeated-measures ANOVA was conducted after transforming the variables to achieve an approximately normal distribution. Differences between variables were considered statistically significant when *p* < 0.05. All statistical analyses were performed using the SAS System for Windows (Statistical Analysis System), version 9.04 (SAS^®^ Institute Inc., 2002–2008, Cary, NC, USA), and R version 4.2.3.

## 3. Results

### 3.1. Participant Characteristics

A total of 100 family caregivers participated in this study, comprising 50 “cases” and 50 “controls”. In total, 100% were female, and they were predominantly mothers. Regarding the chronic conditions of the children, the conditions that were identified include cerebral palsy, autism spectrum disorder, Down syndrome, type 1 diabetes mellitus, and sequelae from prematurity. A detailed characterization is presented in Table 1.

### 3.2. Analysis of Perceived Stress

Regarding the experience of stressful situations in the past 30 days, 40 (80%) caregivers from the case group and 29 (58%) from the control group reported having encountered a stressful situation recently. Among the cited stressful situations, the following reasons were highlighted: the child’s illness, family issues, and school adjustment. A statistically significant difference was observed in the perceived stress scores between the groups. As indicated in Table 2, an increase of 6.24 in the Perceived Stress Scale score is estimated for the case group compared to the control group.

Subsequently, repeated measures ANOVA models were tested, which included, in addition to the group variable, the sociodemographic variables of interest. The significant difference in the perceived stress scores between the groups was maintained. The sociodemographic variables that remained significant, after controlling for group effects, were the family caregiver’s age, household income, and stressful events. According to the estimated effects presented in Table 3, for each additional year in the caregiver’s age, a decrease of 0.2845 in the perceived stress score was expected; for each additional real (currency unit) in household income, a decrease of 0.0005 points in the stress score is anticipated. Regarding the variable “stressful event,” an increase of 3.99 in the perceived stress score is estimated for those who reported experiencing stressful situations in the past 30 days.

### 3.3. Analysis of Salivary Cortisol

**Figure 2:**
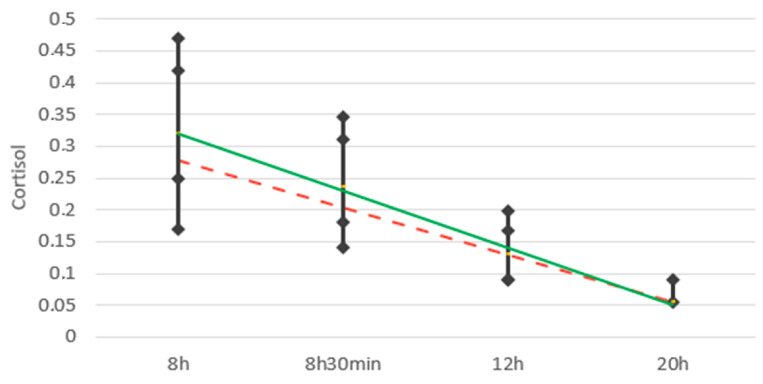
Average cortisol levels throughout the day.

Figure 2 graphically presents the average cortisol levels throughout the day. The dashed red line represents the circadian rhythm of salivary cortisol for the case group, while the solid green line represents the circadian rhythm of salivary cortisol for the control group. It is evident that the circadian rhythm is preserved in both groups, with high cortisol concentrations upon waking and a progressive decrease in the following hours.

Table 4 presents the comparison of daily salivary cortisol production values (AUCG–Cortisol) between the groups based on the Wilcoxon test, identifying a lower total daily cortisol production value among caregivers of children with chronic conditions compared to the control group (*p* = 0.0205). However, in the repeated-measures ANOVA, considering the groups while keeping other sociodemographic variables constant, no difference in AUCG–Cortisol results was observed, although it approached a statistically significant *p*-value (*p* = 0.0572).

Finally, repeated measures ANOVA models were tested to determine the best model for AUCG–Cortisol, incorporating sociodemographic variables and the perceived stress score. There was no significant correlation between daily cortisol production and perceived stress. The variables of a family caregiver’s age and occupation were found to be significant in explaining the behavior of AUCG–Cortisol. According to the estimated coefficients presented in Table 5, an increase of 0.0899 in AUCG–Cortisol is expected among caregivers who are salaried employees compared to those who self-identify as “homemakers.” Regarding the family caregiver’s age, each additional year is expected to result in an increase of 0.0030 in the AUCG–Cortisol value.

## 4. Discussion

In the present study, the mean perceived stress score among family caregivers of children with chronic conditions was 33.76, while in the control group, it was 27.52, indicating a statistically significant difference. A study conducted in Iran on 250 caregivers of children with disabilities, using the same instrument to assess perceived stress, reported a mean score of 27.88, which is similar to the mean observed among caregivers of healthy children and lower than the perceived stress in the case group [5]. Similarly, a study conducted in Turkey on mothers of children with physical and/or mental disabilities identified a perceived stress mean approximately 10 points lower than that observed in the current study [17].

However, two other investigations conducted in the Brazilian context corroborated the findings of the present study. For instance, a study carried out on children with conditions affecting neuromotor and sensory development reported an average score of 30.56 points using the Perceived Stress Scale [18]. Additionally, research involving family caregivers of children and adolescents hospitalized for cancer treatment found that 41% of participants experienced high perceived stress, with scores ranging from 30 to 43 points [19]. A possible hypothesis to explain the higher caregiver stress scores in Brazilian studies may include sociocultural differences between countries.

The statistically significant difference revealed between the groups in the perceived stress score can be explained by the emotional and physical burden associated with caring for a child with a chronic condition. According to a study conducted on caregivers of children with intellectual developmental disorders, there is a positive correlation between the increase in perceived stress levels and the caregiving burden [20].

Among the sociodemographic variables that maintained a significant relationship with perceived stress, controlling for group effects, caregiver age and income stood out. Younger caregivers and those with lower income levels showed higher perceived stress scores, regardless of the group they belonged to. This finding partially aligns with a recent publication that analyzed sociodemographic factors related to the stress of mothers of children with special health needs, in which a negative association between maternal age and perceived stress was observed; however, family income lost significance in the multiple linear regression analysis and was not included in the final model [18].

The relationship between income and stress can be explained by the greater financial capacity of individuals to access resources that can help mitigate stress, such as professional assistance, support services and leisure opportunities. Furthermore, higher income facilitates access to specialized therapeutic technologies and services, alleviating the emotional and physical burden on caregivers and providing security for the family as a whole, creating a more favorable environment for well-being and quality of life [21].

In the present study, lower daily total cortisol production was observed among participants in the “case” group. Lower cortisol levels among family caregivers of children with chronic conditions were identified in studies comparing this biomarker with parents of healthy children [22,23]. The hypothesis of decreased cortisol activity among family caregivers of children with chronic health conditions aligns with the idea of a protective response in individuals experiencing chronic stress. In fact, low cortisol levels have also been reported among individuals with post-traumatic stress disorder (PTSD) [23]. Prolonged exposure to stress (both mental and physical) leads to the decreased sensitivity of the HPA axis, resulting in reduced cortisol production by the adrenal glands [24].

Statistical tests indicated a significant relationship between caregiver age and daily cortisol production. According to a systematic review that explored the effects of caring for a child with congenital, chromosomal, or genetic disease on the regulation or dysregulation of the HPA axis, stress levels can be critically elevated immediately after diagnosis as daily routines change, new habits are adopted and caregivers face many uncertainties. However, stress may decrease over time as caregivers adapt to their new reality [7].

The results also indicated an increase in daily cortisol production among caregivers who are employed compared to those who identify as “homemakers.” Employment can have a positive impact on maternal mental health as work may serve as a form of respite for parents, offering a space where they can restore or replenish their energy and thus act as a protective factor. On the other hand, working women also have to manage household tasks and care for their children, which can amplify stress [25].

The present study did not identify a significant correlation between cortisol and perceived stress, corroborating other studies on the subject. A study that analyzed the association between cortisol levels, stress, depression, and anxiety among 93 mothers and 53 fathers of children with different chronic conditions concluded that total cortisol was statistically significantly associated with anxiety and depression (*p* = 0.004; *p* = 0.034, respectively), while its association with overall stress did not reach statistical significance (*p* = 0.088) [26]. Similarly, an intervention study that included a service dog in the family of 98 primary caregivers of children with ASD found a significant reduction in perceived stress but no change in cortisol levels [27]. In contrast, research involving mothers of children with autism and other developmental disabilities revealed that mothers reporting high perceived stress had reduced cortisol levels, while those with low perceived stress exhibited higher cortisol levels [28].

Despite the contradictory results, the incorporation of biomarkers with self-report measures in research reflects an acknowledgment of the importance of adopting a complex system perspective to understand the dynamic relationships between stress, social contexts, and family caregiving outcomes [10,29].

## 5. Conclusions

The validity of these findings is noteworthy, especially given the support found in the literature. Among the three hypotheses initially proposed, hypotheses 1 and 3 were confirmed. Specifically, family caregivers of children with chronic health conditions experience higher levels of perceived stress, and sociodemographic variables are related to both perceived stress levels and salivary cortisol. However, in the present study, lower total daily cortisol production was observed among family caregivers in the ‘case’ group, contrary to the prediction in hypothesis 2.

In addition, it is important to highlight the limitation of this study’s cross-sectional design, and it is essential to invest in future research that analyzes the stress process over time, as well as the longitudinal impact on the HPA axis of family caregivers.

This investigation presents elements that can inform the planning of personalized interventions considering the social and economic context of families. It is recommended to prioritize health and actions that promote well-being among younger individuals and those with lower incomes. Furthermore, these findings have the potential to create rich opportunities for future intervention research.

## Figures and Tables

**Table 1:** Sociodemographic characteristics of participants.

Variables	Case	Control
	Mean (SD)	Mean (SD)
Family caregiver’s age	38.22 (6.88)	38.16 (6,08)
Child’s age	6.24 (2.76)	6.06 (2,83)
Household income (BRL)	4.376 (3.776)	9.824 (6.722)
	*n* (%)	*n* (%)
Education level		
Elementary school	31(62)	18 (36)
Higher education	19 (38)	32 (64)
Marital status		
With partner	41 (82)	46 (92)
No partner	9 (18)	4 (8)
Occupation		
Self-employed	9 (18)	9 (18)
Salaried employee	15 (30)	33 (66)
Homemaker	24 (48)	6 (12)
Unemployed	2 (4)	2 (4)
Children’s Chronic Conditions		
Cerebral palsy	7 (14)	-
Autism spectrum disorder	21(42)	-
Down syndrome	7 (14)	-
Type 1 diabetes mellitus	4 (8)	-
Sequelae from prematurity	11 (22)	-

Table created by the authors.

**Table 2:** Fixed effect test of perceived stress according to groups.

Perceived Stress	Mean (Sd)	Estimate	*p*-Value
Case	33.76 (6.07)	6.2400	<0.0001
Control	27.52 (8.09)	Reference	-

Table created by the authors.

**Table 3:** Fixed effect test of perceived stress according to group and sociodemographic variables of interest.

Variables	Estimate	*p*-Value
Family caregiver’s age	−0.2845	0.0098
Household income	−0.0005	0.0003
Stressful event		
Yes	3.9901	0.0115
No	Reference	-

Table created by the authors.

**Table 4:** Comparison of daily salivary cortisol production (AUCG–cortisol) between the groups.

AUCG–Cortisol	Mean (Sd)	Median	Minimum–Maximum	*p*-Value
			0.0205	
Case	98.14 (45.75)	87.28	39.12–243.60	
Control	121.41 (70.75)	105.25	46.89–355.50	

Table created by the authors.

**Table 5:** ANOVA of AUCG–cortisol in relation to the family caregiver’s age and occupation.

Variables	Estimate	*p*-Value
Family caregiver’s age	0.0030	0.0405
Occupation		-
Self-employed	0.0910	0.1178
Salaried employee	0.0899	0.0448
Unemployed	0.1927	0.0598
Homemaker	Reference	-

## Data Availability

The original contributions presented in the study are included in the article, further inquiries can be directed to the corresponding author.
